# Supplementary material for: Analyzing Genome-Wide Association Studies with an FDR Controlling Modification of the Bayesian Information Criterion
Source: PLoS One. 2014 Jul 25;9(7):e103322. doi: 10.1371/journal.pone.0103322 (PMC4111553; doi:10.1371/journal.pone.0103322)
Supplement: Table S1 — Summary of analysis results for Bipolar Disorder. (PDF) [file pone.0103322.s001.pdf]

Table S1: Summary of analysis results for **Bipolar Disorder**. The first column gives the reference SNP ID number from dbSNP, followed by the chromosome (Chr) and the position (Pos). The column Gene contains information about the closest lying gene. The final four columns have bullets whenever a SNP was detected by MOSGWA (M), Hlasso (HL), GWASelect with parameter $\xi = 0.3$ (G3) or $\xi = 0.2$ (G2).

dbSNP	Chr	Pos	Gene	M	HL	G3	G2
rs2953145	2	241515596	RNPEPL1				•
rs4627791	3	32347824	CMTM8				•
rs715891	5	145986083	PPP2R2B				•
rs10993706	9	93602967	SYK				•
rs11622475	14	104509076	TDRD9				•
rs2576561	16	55470974	MMP2				•
rs7243929	18	8455102	PTPRM				•
rs12980129	19	22908911	ZNF99				•
rs2837588	21	41748059	DSCAM	•	•	•	•
