# Supplementary material for: Analyzing Genome-Wide Association Studies with an FDR Controlling Modification of the Bayesian Information Criterion
Source: PLoS One. 2014 Jul 25;9(7):e103322. doi: 10.1371/journal.pone.0103322 (PMC4111553; doi:10.1371/journal.pone.0103322)
Supplement: Table S2 — Summary of analysis results for Coronary artery disease. (PDF) [file pone.0103322.s002.pdf]

Table S2: Summary of analysis results for **Coronary artery disease**. The first column gives the reference SNP ID number from dbSNP, followed by the chromosome (Chr) and the position (Pos). The column Gene contains information about the closest lying gene. The following four columns have bullets whenever a SNP was detected by MOSGWA (M), Hlasso (HL), GWASelect with parameter $\xi = 0.3$ (G3) or $\xi = 0.2$ (G2). The crosses in the last column (W) indicate the region which was reported in the original WTCCC publication.

dbSNP	Chr	Pos	Gene	M	HL	G3	G2	W
rs906766	3	150811294	MED12L	•	•	•	•	
rs10965219	9	22053687	CDKN2B-AS		•	•	•	x
rs6475606	9	22081850	CDKN2B-AS				•	x
rs1333049	9	22125503	DMRTA1	•	•	•	•	x
