# Supplementary material for: Analyzing Genome-Wide Association Studies with an FDR Controlling Modification of the Bayesian Information Criterion
Source: PLoS One. 2014 Jul 25;9(7):e103322. doi: 10.1371/journal.pone.0103322 (PMC4111553; doi:10.1371/journal.pone.0103322)
Supplement: Table S3 — Summary of analysis results for Hypertension. (PDF) [file pone.0103322.s003.pdf]

Table S3: Summary of analysis results for **Hypertension**. The first column gives the reference SNP ID number from dbSNP, followed by the chromosome (Chr) and the position (Pos). The column Gene contains information about the closest lying gene. The final four columns have bullets whenever a SNP was detected by MOSGWA (M), Hlasso (HL), GWASelect with parameter $\xi = 0.3$ (G3) or $\xi = 0.2$ (G2).

dbSNP	Chr	Pos	Gene	M	HL	G3	G2
rs7961152	12	24981611	BCAT1				•
rs921535	15	74111343	TBC1D21				•
rs16945811	17	1294614	YWHAE	•	•	•	•
rs1022684	20	18487506	SEC23B				•
