# Supplementary material for: Analyzing Genome-Wide Association Studies with an FDR Controlling Modification of the Bayesian Information Criterion
Source: PLoS One. 2014 Jul 25;9(7):e103322. doi: 10.1371/journal.pone.0103322 (PMC4111553; doi:10.1371/journal.pone.0103322)
Supplement: Table S4 — Summary of analysis results for Crohn's disease. (PDF) [file pone.0103322.s004.pdf]

**Table S4:** Summary of analysis results for **Crohn's disease**. The first column gives the reference SNP ID number from dbSNP, followed by the chromosome (Chr) and the position (Pos). The column Gene contains information about the closest lying gene. The following four columns have bullets whenever a SNP was detected by MOSGWA (M), Hlasso (HL), GWASelect with parameter $\xi = 0.3$ (G3) or $\xi = 0.2$ (G2). The crosses in the last column (W) indicate the regions which were reported in the original WTCCC publication. In yellow we highlight those SNPs which were exclusively detected by MOSGWA.

| dbSNP | Chr | Pos | Gene | M | HL | G3 | G2 | W |
|---|---|---|---|---|---|---|---|---|
| rs17375018 | 1 | 67655147 | IL23R CD25 | | | | • | x |
| rs11805303 | 1 | 67675516 | IL23R | • | • | • | • | x |
| rs10489629 | 1 | 67688349 | IL23R | | • | • | • | x |
| rs41396545 | 1 | 67689608 | IL23R | | | • | • | x |
| rs12119179 | 1 | 67747415 | IL12RB2 | | | • | • | x |
| rs12035082 | 1 | 172898377 | TNFSF18 | • | | | | |
| rs10210302 | 2 | 234158839 | ATG16L1 | • | • | • | • | x |
| rs11718165 | 3 | 49696797 | BSN | • | | | | x |
| rs7726744 | 5 | 40343276 | PTGER4 | | | | • | x |
| rs12658567 | 5 | 40391932 | PTGER4 | • | | | | x |
| rs16869934 | 5 | 40397352 | PTGER4 | | • | | | x |
| rs17234657 | 5 | 40401509 | PTGER4 | • | • | • | • | x |
| rs9292777 | 5 | 40437948 | PTGER4 | | • | • | • | x |
| rs11957215 | 5 | 40445681 | PTGER4 | | | • | • | x |
| rs1505992 | 5 | 40498577 | PTGER4 | | | • | • | x |
| rs11957134 | 5 | 150230950 | ZNF300 | | • | | | x |
| rs1000113 | 5 | 150240076 | ZNF300 | • | | | | x |
| rs9405639 | 6 | 3419149 | SLC22A23 | • | | | | |
| rs6908425 | 6 | 20728731 | CDKAL1 | • | | | | |
| rs4263839 | 9 | 117566440 | TNFSF15 | • | | | | |
| rs10761659 | 10 | 64445564 | ZNF365 | • | • | | • | x |
| rs10883365 | 10 | 101287764 | NKX2-3 | | • | | | x |
| rs10883371 | 10 | 101292455 | NKX2-3 | • | | | | x |
| rs11627513 | 14 | 97539171 | LOC100129345 | • | | | | |
| rs2076756 | 16 | 50756881 | NOD2 | • | • | • | • | x |
| rs7342715 | 16 | 50787483 | CYLD | | • | • | • | x |
| rs2542151 | 18 | 12779947 | PTPN2 | • | • | • | • | x |
| rs41526044 | 20 | 18800670 | SLC24A3 | • | | | | |
| rs2836753 | 21 | 40291187 | PSMG1 | • | | | | |
