# Supplementary material for: Analyzing Genome-Wide Association Studies with an FDR Controlling Modification of the Bayesian Information Criterion
Source: PLoS One. 2014 Jul 25;9(7):e103322. doi: 10.1371/journal.pone.0103322 (PMC4111553; doi:10.1371/journal.pone.0103322)
Supplement: Table S5 — Summary of analysis results for Rheumatoid arthritis. (PDF) [file pone.0103322.s005.pdf]

Table S5: Summary of analysis results for **Rheumatoid arthritis**. The first column gives the reference SNP ID number from dbSNP, followed by the chromosome (Chr) and the position (Pos). The column Gene contains information about the closest lying gene. The following four columns have bullets whenever a SNP was detected by MOSGWA (M), Hlasso (HL), GWASelect with parameter $\xi = 0.3$ (G3) or $\xi = 0.2$ (G2). The crosses in the last column (W) indicate the regions which were reported in the original WTCCC publication. In yellow we highlight those SNPs which were exclusively detected by MOSGWA.

dbSNP	Chr	Pos	Gene	M	HL	G3	G2	G1	W
rs6679677	1	114303808	RSBN1	•	•			•	x
rs3132671	6	30178287	TRIM26	•	•				x
rs3099844	6	31448976	MICB		•				x
rs707974	6	31629499	BAT4		•			•	x
rs2075800	6	31777946	HSPA1L					•	x
rs17421624	6	32066177	TNXB					•	x
rs3134926	6	32200147	NOTCH4		•			•	x
rs9267954	6	32213052	NOTCH4					•	x
rs910049	6	32315727	C6orf10					•	x
rs9268418	6	32343686	C6orf10	•	•				x
rs9268560	6	32389512	HLA-DRA		•			•	x
rs9268853	6	32429643	HLA-DRB5		•				x
rs9268858	6	32429758	HLA-DRB5	•					x
rs9272219	6	32602269	HLA-DQA1					•	x
rs2856688	6	32654640	HLA-DQB1	•					x
rs7775228	6	32658079	HLA-DQB1		•			•	x
rs6457617	6	32663851	HLA-DQB1		•	•	•	•	x
rs9275418	6	32670244	HLA-DQB1	•	•			•	x
rs9275572	6	32678999	HLA-DQA2					•	x
rs3128963	6	33055780	HLA-DPB1	•	•				x
rs12536071	7	42428460	GLI3	•					
rs12531052	7	42428629	GLI3	•					
rs2104286	10	6099045	IL2RA	•					
rs1945076	11	106382378	GUCY1A2	•					
