# Supplementary material for: Analyzing Genome-Wide Association Studies with an FDR Controlling Modification of the Bayesian Information Criterion
Source: PLoS One. 2014 Jul 25;9(7):e103322. doi: 10.1371/journal.pone.0103322 (PMC4111553; doi:10.1371/journal.pone.0103322)
Supplement: Table S6 — Summary of analysis results for Type I diabetes. (PDF) [file pone.0103322.s006.pdf]

Table S6: Summary of analysis results for **Type I diabetes**. The first column gives the reference SNP ID number from dbSNP, followed by the chromosome (Chr) and the position (Pos). The column Gene contains information about the closest lying gene. The following four columns have bullets whenever a SNP was detected by MOSGWA (M), Hlasso (HL), GWASelect with parameter $\xi = 0.3$ (G3) or $\xi = 0.2$ (G2). The crosses in the last column (W) indicate the regions which were reported in the original WTCCC publication. In yellow we highlight those SNPs which were exclusively detected by MOSGWA.

dbSNP	Chr	Pos	Gene	M	HL	G3	G2	W
rs6679677	1	114303808	RSBN1	•	•	•	•	x
rs1025039	5	35901857	CAPSL	•				
rs9261376	6	29.8-30.2MB	unknown	•				x
rs16894900	6	29395499	OR11A1		•			x
rs6906897	6	29415636	OR12D3	•				x
rs17508548	6	29563067	GABBR1		•			x
rs9258205	6	29703823	LOC285830	•				x
rs1615251	6	29745761	HCG4		•			x
rs3130531	6	31206616	HLA-C	•	•			x
rs4959068	6	31343844	MICA		•			x
rs2248462	6	31446796	MICB				•	x
rs3131631	6	31484683	MICB		•	•	•	x
rs9348876	6	31575276	NCR3		•			x
rs376510	6	31688200	LY6G6C	•				x
rs707915	6	31710968	MSH5		•			x
rs2763979	6	31794592	HSPA1B		•	•	•	x
rs492899	6	31933518	SKIV2L		•		•	x
rs408359	6	32141883	AGPAT1	•				x
rs3129900	6	32305979	C6orf10				•	x
rs3129933	6	32336161	C6orf10	•	•	•	•	x
rs3129934	6	32336187	C6orf10	•		•	•	x
rs9391858	6	32341398	C6orf10	•				x
rs2894254	6	32345689	C6orf10	•				x
rs3135377	6	32385399	HLA-DRA			•	•	x
rs3135393	6	32408842	HLA-DRA				•	x
rs9268877	6	32431147	HLA-DRB5	•	•		•	x
rs4530903	6	32581889	HLA-DQA1	•	•		•	x
rs9272346	6	32604372	HLA-DQA1				•	x
rs9272723	6	32609427	HLA-DQA1		•	•	•	x
s9273363	6	32626272	HLA-DQB1	•	•	•	•	x
rs35120848	6	32670495	HLA-DQB1		•	•	•	x
rs2227127	6	32711782	HLA-DQA2		•	•	•	x
rs2071474	6	32782582	HLA-DOB				•	x
rs241427	6	32804414	TAP2			•	•	x
rs3101942	6	32870057	LOC100294145		•	•	•	x
rs3116985	6	33100590	HLA-DPB2	•				x
rs7382464	6	33150268	COL11A2	•				x
rs6928921	6	85062826	KIAA1009	•				
rs2666236	10	33418872	NRP1	•				
rs11171739	12	56470625	ERBB3	•	•			x
rs17696736	12	112486818	NAA25	•	•			x
rs7157296	14	50566882	C14orf138	•				
rs12924729	16	11187783	CLEC16A	•				x
rs2542151	18	12779947	PTPN2	•				
rs41384747	18	16913046	unknown	•				
