# Supplementary material for: Analyzing Genome-Wide Association Studies with an FDR Controlling Modification of the Bayesian Information Criterion
Source: PLoS One. 2014 Jul 25;9(7):e103322. doi: 10.1371/journal.pone.0103322 (PMC4111553; doi:10.1371/journal.pone.0103322)
Supplement: Table S7 — Summary of analysis results for Type II diabetes. (PDF) [file pone.0103322.s007.pdf]

Table S7: Summary of analysis results for **Type II diabetes**. The first column gives the reference SNP ID number from dbSNP, followed by the chromosome (Chr) and the position (Pos). The column Gene contains information about the closest lying gene. The following four columns have bullets whenever a SNP was detected by MOSGWA (M), Hlasso (HL), GWASelect with parameter $\xi = 0.3$ (G3) or $\xi = 0.2$ (G2). The crosses in the last column (W) indicate the region which was reported in the original WTCCC publication.

dbSNP	Chr	Pos	Gene	M	HL	G3	G2	W
rs9465871	6	20717255	CDKAL1				•	x
rs7917983	10	114732882	TCF7L2				•	x
rs7901275	10	114732906	TCF7L2				•	x
rs4074720	10	114748497	TCF7L2		•			x
rs7901695	10	114754088	TCF7L2		•	•	•	x
rs4506565	10	114756041	TCF7L2	•		•	•	x
rs7077039	10	114789077	TCF7L2			•	•	x
rs7961581	12	71663102	TSPAN8				•	
rs7193144	16	53810686	FTO		•			x
rs8050136	16	53816275	FTO	•		•	•	x
